# Dignity, Personhood, or Sacred Selves? Complicating Medical Literature and Caregiver Narratives in Dementia Care

**DOI:** 10.1002/hast.4994

**Published:** 2025-09-18

**Authors:** Cindy L. Cain

**Keywords:** sacred selves, dignity, personhood, dementia, person centered, social context, interaction, clinical ethics

## Abstract

Perceptions of people living with dementia are shaped by a variety of “narratives” produced by medical authorities, family members of people living with dementia, and paid care workers. Narratives often define how a person living with dementia should be treated, with a focus on dignity and personhood. Using data from published medical literature, a discussion board for family caregivers, and participant observation of a memory‐care unit of a long‐term care facility, this paper compares varying narratives about dementia. The medical literature centers dignity and personhood. However, family members problematize dignity in their constructions of personhood. Meanwhile, paid care workers’ practices complicate both dignity and personhood. This paper argues that we can use sociologist Erving Goffman's concept of *sacred selves* to overcome the limitations of extant narratives and improve care for people living with dementia.

Narratives about the experiences, abilities, limitations, needs, and desires of people living with dementia come from many places. However, with the medicalization of dementia—or the process by which cognitive changes become defined as medical problems rather than social problems—medical authorities become especially influential in shaping cultural ideas about this condition. Medical authorities’ ways of constructing dementia do more than just reflect cultural meanings; they are part of an active process of producing and refining meaning over time.

Although it is clear that dementia has been medicalized, medical authorities are not the only groups that are engaged in constructing narratives about people living with dementia. Caregivers, whether they be family members or paid caregivers (or both), have first‐hand experiences with dementia and may construct narratives that differ from those of medical authorities. On the whole, medical narratives have a great deal of cultural significance, in part because medical narratives are tied to access to diagnoses, resources, services, and legitimacy. Given this, family caregivers may pull on medical narratives in their own efforts to make sense of what the person living with dementia needs. However, there is not complete overlap between medical and family narratives, and the voices of caregivers provide additional insight into the experiences of living with dementia.

In this essay, I will compare and contrast how medical authorities, family caregivers, and nonmedical paid workers each engage with narratives about quality care for people living with dementia, with special attention to two concepts that have been central to moral philosophy and ethical discussions of dementia: *dignity* and *personhood*. These concepts have become so central, in fact, that they are often part of the lay public discourse as well. But how these concepts are understood and reflected in everyday care practices may differ across groups, making it challenging to clearly delineate what is and is not quality care. I analyze medical literature on dementia, blog posts from the Alzheimer's Association,^1^ and ethnographic observation in a memory‐care unit of a long‐term care facility to demonstrate that caregiving interactions can be interpreted through multiple narratives, illustrating tensions in how we think about (and thus how we teach and evaluate) quality care for people living with dementia.

Examining differences by context also surfaces limitations in the concepts of dignity and personhood. I argue that another concept from microsociology, the *sacred self*, may be a fruitful new lens through which to understand which actions are most aligned with quality care for people living with dementia.

## Dignity and Personhood

Dementia emerged as a distinct condition in the early twentieth century, and the dominant narrative at that time was that a person living with dementia was no longer there, even as their body continued to survive. There was a common sentiment that dementia robbed people of what made them human.^2^ In more recent decades, care providers, scholars, family members, and advocates have pushed for alternative narratives, especially those related to dignity and personhood. Narratives reflecting these concepts are used to assert that people living with dementia are still human and therefore are owed dignity and deserve to be treated as persons. However, when scholars try to delineate which actions are consistent with dignity or personhood, it becomes clear that the terms “dignity” and “personhood” can lack specificity, and the translation to action is not always discernable. Some acts that literature has claimed are “undignified” may be personhood supporting; meanwhile, some attempts to maintain dignity decrease autonomy and the well‐being of people living with dementia, thereby undermining personhood. In both situations, the social context of the act is what determines whether it is conducive to quality care. Below, I will outline the parameters of extant cultural narratives related to dignity and personhood. After discussing some of the tensions in how the terms are used, I propose an alternative narrative that grounds actions in the interactional context and provides more real‐life guidance about how to determine if an act is supportive or not.

## Medical Authorities

To begin my analysis of medical authorities’ narratives, I used the Web of Science database, which aggregates scholarship from a wide range of fields and allows analysis of publication and citation trends over time. The number of articles published on dementia has increased dramatically from the 1990s to today. From 1990‐1999, just under 16,000 articles about dementia were published. The numbers increased in each decade, and from 2020 to early 2023, more than 32,000 articles were published. In the first three years of this decade alone, we have seen a doubling of the number of dementia‐related articles from the entire decade of the 1990s. Because Web of Science aggregates from all scientific disciplines, these articles cover a wide variety of topics, from purely biological and neurological accounts of the disease process to social models of care and recommendations for interventions.

**Figure 1:**
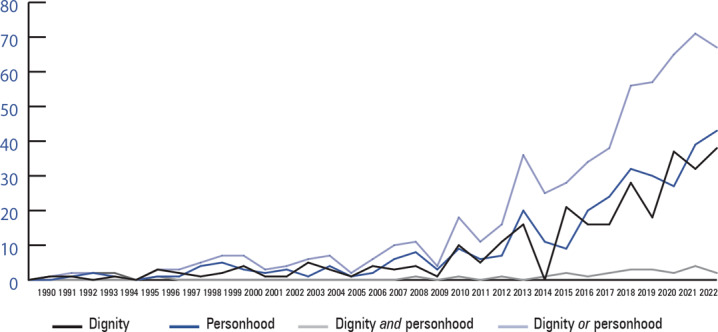
The Number of Scholarly Science Articles about Dementia That Include the Terms “Dignity,” “Personhood,” or Both^1^ 1. This analysis was conducted in August 2023 and was limited to articles written in English.

To go deeper into how medical authorities conceptualize the experience of dementia, I searched these articles for the concepts of dignity and personhood. I started with the full list of articles that Web of Science classified under the topic of “Dementia,” which means that the word “dementia” appeared in each article's title, abstract, or keywords. At the time of data collection, this included just over 134,000 articles written in English. I added the search terms “dignity” and “personhood,” and the search then identified articles among the 134,000 that had either or both of these terms anywhere in them. The use of the two terms follows very similar patterns over time and reflects that the interest in psychosocial dimensions of the experience of dementia grew alongside the growth in dementia scholarship more generally. The line graph (figure 1) shows how these terms correspond to one another over time. The black line indicates articles that use “dignity,” and the dark blue line indicates articles that use the term “personhood.” The light blue line indicates articles that use either term. The gray line is for articles that use both terms. While there is some conceptual overlap across the terms, it is quite rare for them to be used in conjunction. Although more research is needed to know why the terms were not used together, it is possible that this reflects disciplinary differences in preference for terms or that multiple subsets of scholars were thinking about these issues at the same time but were not necessarily in conversation with one another.

Despite the clear increase in the inclusion of either “dignity” or “personhood,” these articles make up a tiny proportion of all articles on dementia: for the 1990s, articles with the concept of dignity or personhood make up only 25 articles out of a total of almost 16,000 published (a rate of 16 per 100,000). Since 2020, that proportion has gone up to 206 articles out of more than 32,000 (a rate of 63 per 100,000). The emphasis on dignity and personhood began rising around 2010 and was still going strong as of August 2023.

Examining publication statistics gives some indication of the prevalence of particular narratives about dementia but does not illustrate the content of those narratives. Case studies, commentaries, and letters published in dominant medical journals can be a source of narrative content. In many of the texts, “dignity” and “personhood” are used to describe what should happen within care, but the definitions of these terms are not spelled out.

Other texts engage in explicit theorizing and offer guidance on how the concepts relate to care practices. One prominent example is an article called “How Are We Going to Live with Alzheimer's Disease?,” written by bioethicist Jason Karlawish and published in *Health Affairs*, a leading health policy journal.^3^ The article argues that Alzheimer disease raises a host of ethical questions. He sets up the question, “How do we live with dignity and quality of life in the face of progressive disability and, ultimately, death?”^4^ Even in the framing of this question, the author is centering the idea of dignity and implies that dementia is a threat to dignity.

In the text, Karlawish seems to define dignity primarily in terms of individuals’ autonomous decision‐making, something that is common in the scientific literature. He asserts that “[a] system of laws, ethics, and social norms grants each of us the freedom to answer this question [“How do we live with dignity and quality of life in the face of progressive disability and, ultimately, death?”] and to respect each other's autonomy to do the same, but patients with Alzheimer's disease face challenges in their ability to participate in this system.”^5^ Because he associates dignity with autonomy and thinks of autonomy as primarily a cognitive process, he argues that dignity is supported when we help people to be able to make the right decisions for themselves, even if they cannot make those decisions completely on their own. This cognitive definition of autonomy and its link to dignity is common in the clinical literature. However, recent bioethics scholarship argues that people (with and without dementia) make decisions in conjunction with one another and that sometimes autonomy is exercised through nonverbal and even noncognitive ways, such as physically using one's body to refuse particular kinds of touch or treatment.^6^

Beyond using the concept of autonomy to define “dignity,” the medical literature often outlines experiences and activities that are thought to be more or less dignified. For people living with mild or moderate dementia, the clinical literature points to loss of previous identity, increases in dependency, and infantilizing or stigmatizing language as aspects of social life that reduce personal dignity.^7^ Recommendations for ensuring dignity often focus on social integration, including avoiding embarrassment or shame, protecting privacy, and providing opportunities for meaningful engagement with others. Markers of dignified behavior, such as wearing appropriate clothing, being presentable, and being treated as a contributing member of a group, are central.^8^

The concept of personhood is rooted in another narrative that is common in the clinical literature and comes from Thomas Kitwood's now classic *Dementia Reconsidered: The Person Comes First*.^9^ In this book, Kitwood argues that the context of care is central to questions of maintaining cognitive functioning and quality of life. He discusses how many care institutions are organized and staffed in ways that further cognitive decline. For example, people without opportunities to engage with others will experience declines quickly, and many care institutions are designed around the basic tasks (such as providing meals, toileting, and bedding) rather than social interaction. But, if we design care systems that focus on relationships, recognition of the personhood of people living with dementia, and the maintenance of connection to the social group, people living with dementia will have better quality of life, and we can slow the progression of the disease.

Early on, the Alzheimer's Association created “Guidelines for Dignity” recommendations,^10^ based on the idea that treating people well meant ensuring their dignity. More recently, the association changed its language to “person‐centered care,” signaling the importance of work like Kitwood's.^11^ This change was critical for getting beyond “the traditional medical model of care that tends to focus on processes, schedules, and staff and organizational needs.”^12^ However, as practiced in many care settings, person‐centered care is often focused on learning biographical details about a person's (former) life, rather than ensuring a current social life and understanding of what constitutes personhood as the former life becomes less relevant. Looking over time, it is clear that medical authorities have devoted a great deal of time and energy to dementia. The psychosocial well‐being of people living with dementia has been a small but influential concept within the medical literature. In narratives concerning the psychosocial well‐being of people with dementia, both dignity and personhood have been concepts that are promoted and have been integrated beyond the clinical boundary, including in advocacy and policy recommendations.

## Family Caregivers

While the medical literature focuses on setting guidelines for what should and should not be included in care for people living with dementia, family members do not always share the same assumptions about quality of care. In this section, I draw on posts from the Alzheimer's Association blog to illustrate how the clinical terms “dignity” and “personhood” are challenged when they are enacted within family life. I selected this blog because of its visibility in this space and its diversity of topics and authors, including family members. I analyzed posts from 2015 to 2020 that were written by family members and included “dignity,” “personhood,” or both in the text. From these posts, we see that family members do not always define quality care in ways that are consistent with the clinical literature.

Treating people living with dementia in ways that could infantilize them has long been considered undignified, and yet the practice continues and is sometimes appreciated by family caregivers. One daughter wrote a thank you to her mother's medical providers, saying, “Whatever role you play in the care of patients with Alzheimer's disease, please know that I see you. I see how you care about and even love her. I hear you lovingly call her ‘honey’ and ‘sweetie’ and laugh with her when she is confusingly silly. You are so important in the life of a patient with Alzheimer's disease. They need you so much. They need you to see them, to see they are just as human as you are. They need kindness and respect. They need you to help maneuver their very confusing days. They need dignity. They need silliness and smiles, compassion and love, patience and hugs. They need you, and their families need you.”^13^ The writer is forwarding the idea of “dignity” in her post, but some of what she includes—pet names, laughing at silliness—contradicts dictates not to infantilize people who have dementia. Other aspects of the post, such as “see they are just as human as you are” are clearly in line with Kitwood's conceptualization of personhood. In this example, the daughter perceives that her mother's personhood is supported by actions that may be considered “undignified.”

For some people living with dementia and their family caregivers, privacy and the ability to control what is shared and with whom is a central component of dignity. Another adult child writes of her mother, “She didn't lose her smile, she didn't lose the love for those around her, but she did lose her dignity. Mom was a very private person and I could count on one hand the times I saw her in her bra and underwear before Alzheimer's, then suddenly I'm changing her diapers. We promised our dad we would take care of her and we kept that promise [*sic*].”^14^ The writer claims that her mother remained the same in some important ways (her smile and her love of others) but that having her bodily processes on display for her children harmed her dignity. It is an open question as to whether the mother felt like her dignity was violated in the moment or if this is a construction the daughter formed through her experiences with her mother before her cognitive limitations. Either way, the linking of privacy and boundaries between public and private was a central component of what this family member thought was dignified, even as it sounds as though her mother's care was person centered. The daughter's sense that the bodily intimacy involved in care stripped her mother of dignity suggests that there was no way to avoid the loss of dignity.

When the concepts of dignity and personhood are imported into family life, they can take on connotations that differ from those in the medical literature. In these examples, concerns about infantilization are downplayed, and protection of privacy becomes an unavoidable attack on dignity, even as both family members discuss care that is personhood supporting. Perhaps family caregivers are less knowledgeable about the debates and developments in thinking about quality care for people living with dementia. Alternatively, perhaps this difference is more about how family histories and relationships create a different kind of care context, with different ways of defining dignity and personhood.

## Care Workers

As the examples above show, black‐and‐white definitions of what is and is not dignified or supportive of personhood are complicated when one considers the acts of care. For both clinicians and family members, the terms “dignity” and “personhood” could lead them to a variety of behaviors, which can make decisions about how to act in interaction difficult. Adding care worker narratives to those of medical authorities and family caregivers provides context that may matter for how dignity and personhood are operationalized. While many paid caregivers working with people with dementia in long‐term care facilities have received dementia‐specific training that emphasizes a medical narrative of disease, the workers have then needed to determine how to translate this narrative into action sanctioned by the organizations where they work. Their care interactions reveal additional moments when the ideas of dignity and personhood become complicated.

The following excerpts come from my ethnographic observations of a memory‐care unit of a long‐term care facility known for its high‐quality practices and committed staff. I observed this unit for three to five hours per week for nine months. I concentrated my observations on interactions between staff members and residents in common areas, such as the dining room or television area. For this analysis, I selected interactions that further problematize our existing definitions of dignity, personhood, and the act of care.^15^

*One of the certified nursing assistants [CNAs] went over to Mr. Michael and started brushing his hair. She didn't ask him before doing it, and he didn't seem to mind. He even leaned into it a bit, indicating that it might feel good. A few minutes later, another CNA walked by and spoke to him by name. He looked at her but didn't respond to her question about what was on his face (a scratch). As she walked away, she spoke to another staff member and asked if he had had a haircut. The CNA said no, but that she had just brushed his hair.*

Mr. Michael's body language indicated that he did not resist the act of hair brushing. The CNAs treated it as an act of affection and as part of their caregiving role. But there are aspects of the encounter that would not fit what the clinical literature defined as dignified care or even personhood supporting. The CNA did not ask for consent, they groomed him in a public area (blurring the public/private divide), and they talked about him as though he was not there.

In another instance, workers attempted to reinforce the independence of residents, which meant carefully monitoring a situation that could become hazardous but allowing it to unfold as long as it remained safe.

*Two of the ladies by the window were talking quietly and seemed to decide to get up and go for a walk. The more mobile one got up and tried to help the other one get up, but she was wedged in, and her walker was on the other side of the table. After several minutes of the first resident trying to move the table and help the other one to her feet, the second resident was finally standing. All the while, the more mobile resident was telling the other one that she didn't need the walker. A nearby CNA watched closely, but did not intervene. Once the less mobile resident started moving, though, the CNA said, “Elaine, get your walker.” It was clear that the CNA was watching all of this and intervened only when the resident was just about to start walking without it. Elaine couldn't figure out who was talking to her and looked around with confusion. The CNA repeated herself several times.*

*Finally, Elaine found the source of the voice and said, “I don't know what. This?” and pointed to a chair nearby.*

*The CNA walked over and grabbed the walker and put it in front of Elaine, saying, “Here. Use this.” Elaine thanked her and commented that it is hard to sit all day and she needs to walk around. The CNA nodded and went back to her table.*

One could interpret the CNA's actions as negligent, and in fact, when I presented this example at a scholarly conference, attendees were outraged that the CNA did not intervene earlier. However, if you view the encounter from the perspective of empowering the resident to be as independent as possible, the CNA's behavior was personhood supporting in that it allowed Eileen to try to solve the problem on her own—something linked to autonomy‐based notions of dignity.

Examples like these indicate that existing narratives about dignity and personhood are still inadequate for conveying the full range of ways that people with dementia can be supported and treated well. While the clinical literature and care guidelines are well‐intentioned, their link to actions is not always clear and sometimes produces unintended effects. Some indignities are affectionate and may be perceived as personhood supporting. Some standards of dignity undermine personhood, as is the case when dignity dictates that one must dress and act in a particular way, even if they do not feel like it. Conformity to abstract principles of dignity and person‐centered care can lose the spirit of personhood that Kitwood put forward. In the final section, I introduce another concept from the micro‐interactionist tradition of sociology—sacred selves—which can help clinicians, family members, and care staff to think about how the interactional context matters for knowing what is supportive of dignity and personhood.

## Sacred Selves

Erving Goffman, an American sociologist from the twentieth century, is credited with a range of theories that seek to understand how social interactions generate a sense of self and a connection to the larger social group. Goffman thought that small moves in the interactive context created one's ability to have a “sacred self,” defined as a self that is individuated, treated as important, and shown deference, as a sacred object may be.^16^ For example, when one enters a room and is greeted by others, it reinforces the individual's sense that they are important and worthy of acknowledgment. The individual then also perceives the others who greeted them favorably, helping fulfill others’ needs to feel sacred as well. In this way, not only are all individuals respected and treated as “sacred,” but the social group as a whole is solidified. Through normal interactional exchanges, we become bound to the group and built up as individuals.

Dementia, as a condition that affects memory, communication, and behavior, can pose a challenge to establishing and maintaining “normal” interactions. And yet, because so much of our interactional activity is habitual, even people with moderate dementia will still often engage in these rituals. Expanding the discussion of quality care to include the lens of sacred selves provides new tools for deciding which actions are more or less supportive of dignity and personhood.

For example, autonomy, which was so important to Karlawish, is a value to seek out, but the distinction between *autonomous* and *nonautonomous* is more complex than it initially seems. In the ethnographic example of the CNA helping Elaine to find her walker, the CNA allowed Elaine to try to complete the task and make her own decisions about how to do so but eventually had to intervene. In giving Elaine space to try, she was also supporting her “sacred self,” allowing her to save face when she didn't understand the difference between a chair and a walker. The CNA did not try to explain that the chair was not a walker. Instead, she just brought over the walker and ignored the mistaken identification. This move is more than just ensuring dignity or treating Elaine as a person. Taking a more limited role in the encounter demonstrates the CNA's respect for Elaine as a sacred member of the social group, worthy of inclusion.

Looking at the infantilizing language in the Alzheimer's Association blog post and the example of the CNA combing Mr. Michael's hair, some may still be uncomfortable with caregivers treating a person with dementia like a child, but it may also be that these kinds of actions are used to convey affection and intimacy. Goffman shows how the use of space between people and whom we allow in our personal space indicate larger social relations. It may be undignified to groom someone in public, but it may also be an important part of supporting the personhood of the person cared for. In fact, part of respecting the sacred self is an acknowledgment that the self can change over time. As dependency increases, perhaps the ways we convey sacredness also change. In this situation, it is not about treating people living with dementia as children but about creating a new narrative that they are elders in need of assistance, which is their due as valued members of society. In fact, many CNAs in this study conveyed exactly that message. They often constructed the people they cared for as precious, important individuals with rich histories and current‐day preferences, even as they also needed assistance.

The concepts of dignity and personhood currently do not allow for this change over time—at least in the ways they are operationalized into expectations for activities. When actions are determined to be dignified or not, there is no room for those actions to change in meaning. For example, if breaching privacy is thought to be undignified, as one of the daughters described in the Alzheimer's Association blog, it will always be undignified to perform intimate bodily care such as changing adult briefs, no matter how much this is needed. Likewise, if person‐centered care is framed in terms of “knowing” a person but that knowing is couched in their prior lives, there is no room to change to reflect the person who is currently living with dementia and who may have differing thoughts, feelings, and sense of self.

These brief examples illustrate how a momentary care encounter can include multiple components, which may vary in terms of the meanings they produce about quality care. While our existing concepts of dignity and personhood are critical for centering the subjective experiences of people living with dementia, they are also limited in their understanding of how the care and interactional contexts matter. The sacred‐selves lens offers another perspective on what happens within interactions and allows the definitions of quality care to change alongside the status of the person living with dementia. This lens can also guide decisions about how to organize clinical and custodial care settings to provide the most opportunities to treat one another as sacred members of the group. Simple changes, like speaking directly to the person living with dementia (instead of their family members) have already been suggested and should be prioritized. Additional changes include honoring verbal and nonverbal communications from the person living with dementia, even when they do not always make sense or comport with expectations. Clinicians and caregivers also need to provide space for needs and the self to change over time. Through interactions that support the sacred selves of people living with dementia, both dignity and person‐centered care will be improved, while also allowing adaptation and individuation.
